# Effects of sulfur amino acid supplementation on broiler chickens exposed to acute and chronic cyclic heat stress

**DOI:** 10.1016/j.psj.2022.101952

**Published:** 2022-05-06

**Authors:** A.H. Sarsour, M.E. Persia

**Affiliations:** Virginia Tech, Blacksburg, VA 24061, USA

**Keywords:** sulfur amino acids, broilers, heat stress, intestinal permeability, oxidative stress

## Abstract

Chronic heat stress can result in oxidative damage from increased reactive oxygen species. One proposed method to alleviate the chronic effects of HS is the supplementation of sulfur amino acids (**SAA**) which can be metabolized to glutathione, an important antioxidant. Therefore, the objective of this experiment was to determine the effects of dietary SAA content on broiler chickens exposed to HS from 28 to 35 d on broiler performance, body temperature, intestinal permeability, and oxidative status. Four experimental treatments were arranged as a 2 × 2 factorial consisting of HS (6 h at 33.3°C followed by 18 h at 27.8°C from 28 to 35 d of age) and Thermoneutral (TN- 22.2°C continuously from 28 to 35 d) and 2 dietary concentrations of SAA formulated at 100% (0.95, 0.87, and 0.80% for starter, grower, and finisher diets) or 130% SAA (1.24, 1.13, and 1.04% for starter, grower, and finisher diets). A total of 648-day-old, male Ross 708 chicks were placed in 36 pens with 18 chicks/pen and 9 replicates per treatment. Data were analyzed as a 2 × 2 factorial in JMP 14 (*P* ≤ 0.05). No interaction effects were observed on broiler live performance (*P* > 0.05). As expected, HS reduced BWG by 92 g and increased FCR by 11 points from 28 to 35 d of age compared to TN, respectively (*P* ≤ 0.05). The supplementation of SAA had no effect on live performance (*P* > 0.05). Cloacal temperatures were increased by 1.7, 1.4, and 1.2°C with HS at 28, 31, and 35 d compared to TN, respectively (*P* ≤ 0.05) and dietary SAA did not alter cloacal temperatures. At 28 d of age, supplementation of SAA to birds exposed to HS interacted as serum FITC-dextran (an indicator of intestinal permeability) was reduced to that of the TN group (*P* ≤ 0.05). The interaction was lost at 31 d, but HS still increased intestinal permeability (*P* ≤ 0.05). By 35 d, broilers were able to adapt to the HS conditions and intestinal permeability was unaffected (*P* > 0.05). Potential oxidative damage was reduced by increased SAA supplementation as indicated by an improvement in the reduced glutathione to oxidized glutathione ratio of 5 and 45 % at 28 (*P* = 0.08) and 35 d (*P* ≤ 0.05). These data suggest that intestinal permeability is compromised initially and to at least three d of heat exposure before the bird can adjust. However, oxidative damage in the liver of broilers exposed to HS is more chronic, building over the entire 7 d HS period and increased dietary SAA might have some protective effects on both broiler intestinal permeability and oxidative stress responses to HS.

## INTRODUCTION

Genetic selection has increased both the growth rate and efficiency of broilers over the last 60 yr contributing to over 400% improvement in growth rates with a 50% improvement in feed and water efficiency (Zuidhof et al., 2014). As a result of this increased growth rate, there is an associated increase in metabolic heat production with these faster growing broilers which will reduce the bird's ability to mitigate heat stress when subjected to elevated temperatures (Nascimento et al., 2017). The first mechanism of heat mitigation includes posture changes to decrease feather coverage and insulation, as well as digging in the litter to find a cooler surface for heat loss by conduction (Mack et al., 2013). Once passive or minimally active methods are exhausted, birds will use evaporative heat loss through panting to cool core body temperatures (Mack et al., 2013).

A previous heat stress (**HS**) experiment using a pair feeding strategy, indicated that approximately 63% of the growth depression in heat stressed chickens can be attributed to reduced feed intake (Dale and Fuller, 1979). Although the majority of body weight gain loss is attributed to reduced feed intake, a third of the weight gain loss could be due to physiological responses. One of the physiological responses that can reduce growth rate during HS is intestinal permeability. Heat stress can increase intestinal permeability by impairing the tight junction proteins that form a barrier between enterocytes (Quinteiro-Filho et al., 2010). The breakdown of tight junction proteins can result in leaking of bacterial metabolites or bacteria into circulation resulting in reduced performance or potential for disease (Wu et al., 2018). Tight junction leaking has been hypothesized to occur as the birds reduce gastrointestinal blood flow diverting blood flow to peripheral tissue as a mechanism to increase heat loss. This reduced gastrointestinal blood flow can result in reduced nutrients and oxygen to the intestinal tract causing intestinal damage (Lambert, 2009). Second, the reduced blood flow can result in a hypoxic state in the intestine resulting in a downregulation of tight junction protein expression and increased intestinal permeability (Tabler et al., 2020).

In addition to increased intestinal permeability, HS can also result in over production of reactive oxygen species (**ROS**). Reactive oxygen species are natural byproducts of cellular oxidative metabolism generated in the mitochondria (Turrens, 2003). The metabolic response to HS in the birds results in an increase in energy demand within the body resulting in an increased production of ROS (Yang et al., 2010). Heat stress can cause damage to the mitochondria resulting in dysfunction of the electron transport chain further increasing ROS production (Mujahid et al., 2005). Excess generation of ROS can oxidize proteins and lipids during HS resulting in the production of more radicals that can further damage cells (Benzie, 1996). Normal ROS levels are maintained in cells with the help of antioxidant enzymes produced endogenously within the body such as superoxide dismutase, catalase, and glutathione peroxidase (Yang et al., 2010). Glutathione plays a direct role in mitigation of peroxides by transferring 2 hydrogen atoms resulting in the formation of a stable water molecule. Oxidized glutathione can be recycled back to reduced glutathione by the donation of 2 hydrogen atoms from Nicotinamide adenine dinucleotide phosphate (**NADPH**) catalyzed by the enzyme glutathione reductase. During oxidative stress, there is a rapid and substantial decline in the glutathione level in the body, so finding strategies to increase glutathione will potentially improve the antioxidant status maintaining the balance of ROS and antioxidants (Willemsen et al., 2011). Reduced glutathione to oxidized glutathione ratio (rGSH:GSSG) is often used to determine redox status as under normal conditions most of the glutathione would be in the rGSH form (Owen and Butterfield, 2010). Glutathione is synthesized in the liver of birds from the amino acid cysteine that alone with methionine constitutes the sulfur amino acids.

Methionine and cysteine are essential to poultry diets as they are used for muscle development and growth (Garcia and Batal, 2005). However, during HS, additional supplementation of SAA might reduce the negative effects of elevated temperatures on broilers. Previous research in piglets has shown that supplementation of methionine can result in a reduction in intestinal permeability due to the increase in expression of tight junction proteins (Chen et al., 2014). One of the mechanisms for SAA improving intestinal permeability is through the reduction in ROS species which can damage cells and DNA within the body. The other mechanism is through the production of polyamines which methionine acts as the primary donor for their synthesis. Polyamines are important in protecting the intestine as well as the tight junction proteins and the result of polyamine depletion would result in the disruption of the barrier function (Guo et al., 2005). The effect of SAA on intestinal permeability has not been investigated in poultry under heat stress; however, in an in-vitro experiment Caco-2 cells were challenged with hydrogen peroxide resulting in an impairment of epithelial barrier function that was ameliorated with supplemental methionine (Martin-Venegas et al., 2013). Previous research has also reported that supplementation of SAA to birds under HS resulted in reduced ROS production, and an increase in antioxidant activity in quail compared to similar quail held under thermoneutral (**TN**) conditions (Del Vesco et al., 2014). Therefore, the objective of the current experiment was to investigate the effects of SAA supplementation on broiler chickens exposed to a cyclic heat stress on broiler performance, cloacal temperature, panting, intestinal permeability, and oxidative stress**.**

## MATERIALS AND METHODS

### Diet Formulation and Production

The corn, soybean meal, DDGS, and poultry byproduct meal used in this experiment were analyzed for amino acid concentrations by wet chemistry (AOAC method 982.03) prior to dietary formulation to generate a control and control plus 30% SAA diets. Broiler diets were formulated using a phase feeding approach including a 0 to 11 d starter, 11 to 21 d grower, and 21 to 35 d finisher diets (Table 1) to meet breeder nutrient recommendations (Aviagen, 2018). A basal diet approach was used where common ingredients were mixed for each dietary phase before being equally split to generate 2 experimental diets. The diets formulated to 100% of the SAA requirement contained 0.95, 0.87, and 0.80% while the diets formulated to 130% of the requirement contained 1.24, 1.13, and 1.04% SAA for the starter, grower, and finisher diets, respectively. Experimental diets were analyzed for amino acid content using wet chemistry (AOAC method 982.03).

**Table 1:** Formulation and nutrient profile of experimental diets for starter (0–11 d), grower (11–21 d), and finisher (21–35 d) diets fed to Ross 708 broilers exposed to elevated environmental temperatures from 28 to 35 d.^1^

Ingredient	Starter		Grower		Finisher	
	^____________________________________^ (%) ^____________________________________^					
Corn	58.28		60.39		64.98	
Soybean meal	33.39		29.66		23.59	
Poultry byproduct meal	2.00		5.00		6.00	
Soy oil	0.68		1.82		2.63	
Sodium chloride	0.18		0.17		0.16	
Sodium-bicarbonate	0.20		0.20		0.20	
DL-Methionine	0.33		0.27		0.24	
L-Lysine	0.22		0.13		0.18	
L-Threonine	0.08		0.03		0.04	
Limestone	0.92		0.86		0.80	
Dicalcium phosphate	0.97		0.86		0.56	
Phytase^2^	0.01		0.01		0.01	
Choline chloride	0.10		0.10		0.10	
V & M premix^3^	0.63		0.52		0.52	

Formulated nutrient	Starter		Grower		Finisher	
	100%	130%	100%	130%	100%	130%
	^______________________________________________^ (%) ^______________________________________________^					
Crude protein^4^	22.8 (22.7)	23.0 (22.9)	21.6 (21.4)	21.8 (21.5)	19.7 (19.4)	19.9 (19.5)
ME, kcal/kg	3,000	3,000	3,100	3,100	3,200	3,200
Calcium	0.90	0.90	0.87	0.87	0.79	0.79
Nonphytate P	0.45	0.45	0.44	0.44	0.40	0.40
Fat	3.90	3.87	5.16	5.14	6.17	6.15
Digestible Met	0.62 (0.62)	0.91 (0.69)	0.55 (0.56)	0.81 (0.77)	0.51 (0.47)	0.75 (0.65)
Digestible Cys	0.33 (0.43)	0.33 (0.43)	0.32 (0.41)	0.32 (0.41)	0.29 (0.34)	0.29 (0.34)
Digestible Met+Cys	0.95 (1.05)	1.24 (1.12)	0.87 (0.97)	1.13 (1.18)	0.80 (0.81)	1.04 (0.99)
Digestible Lys	1.28 (1.25)	1.28 (1.25)	1.15 (1.13)	1.15 (1.13)	1.06 (1.13)	1.06 (1.13)
Digestible Thr	0.86 (0.81)	0.86 (0.81)	0.77 (0.72)	0.77 (0.72)	0.71 (0.72)	0.71 (0.72)
Digestible His	0.56	0.56	0.53	0.53	0.48	0.48
Digestible Trp	0.23	0.23	0.22	0.22	0.19	0.19
Digestible Arg	1.39	1.39	1.31	1.31	1.17	1.17
Digestible Iso	0.85	0.85	0.80	0.80	0.71	0.71
Digestible Val	0.94	0.94	0.90	0.90	0.81	0.81

1 Diets were formulated on a digestible amino acid basis and a calculated digestible amino acid value based on total analyzed amino acid analysis is presented within the (). Analyzed total amino acid values were converted to digestible amino acid values using AminoDat Software (Version 5, 2016) by multiplying the digestibility coefficient of each ingredient by the amount of amino acid provided by that ingredient in the diet.

2 Quantum blue (500 FTU/kg) was formulated to provide 0.10% of calcium and nonphytate phosphorus.

3 Vitamin and mineral premix Provided per kg of diet: vitamin A, 4,403 IU; vitamin D3, 1,457 ICU; vitamin E, 1.10 IU; menadione, 0.77 mg; vitamin B12, 4.40 μg; choline, 254.79 mg; niacin, 13.21 mg; pantothenic acid, 4.05 mg; riboflavin, 2.75 mg; Cu, 2.70 mg; Fe, 33.75 mg; I, 0.67 mg; Mn, 42.90 mg; Zn, 32.50 mg; Co, 0.17 mg.

4 Formulated crude protein values with combustion determined crude protein values reported in ().

### Experiment Design and Broiler Management

All animal procedures were approved by the Institutional Animal Care and Use Committee at Virginia Tech (Blacksburg, VA). Treatments were arranged as a 2 × 2 factorial with temperature: HS (6 h at 33.3°C followed by 18 h at 27.8°C from 28 to 35 d of age) and TN (22.2°C continuously from 28 to 35 d) and 2 dietary concentrations of SAA (0.95, 0.87, and 0.80% or 1.24, 1.13, and 1.04% for starter, grower, and finisher diets, respectively) as the 2 factors. In total, 648 male Ross 708 broiler chicks were allotted to the 4 treatments with 9 replicate pens of 18 broilers in 2 different rooms. Broilers were provided ad libitum access to experimental feed and water. Broilers were fed starter diets in pelleted and crumbled form and pelleted diets for grower and finisher diets. Temperature was maintained according to breeder specifications based on the bird age starting at 30°C at placement to 22°C at 28 d of age (Aviagen, 2018). The TN room was maintained at 22°C continuously from 28 to 35 d. The HS room was adjusted to 33.3°C for 6 h followed by 18 h at 27.8°C and continued in this cyclic fashion daily from 28 to 35 d of age. The room temperature was able to reach the target temperature within 20 min of heat exposure. Continuous lighting was provided from 0 to 3 d of age, then the lighting was adjusted to provide 20 h of light and 4 h of darkness (02:00 till 06:00) from 3 to 35 d of age according to the commercial management guide. Heat stress was initiated 2 h after the lights turned on from 28 to 35 d of age. Health checks occurred at least twice daily when mortality was noted it was removed from the pen, weighed, and recorded.

### Broiler Performance and Body Composition

Birds were weighed individual on d 0, 11, 21, 28, and 35 when diet phases changed or HS was initiated and body weight gain was determined over the 0 to 28 d pre-HS period, the 28 to 35 d HS period and the entire 0 to 35 d period as the difference between final and initial body weights. Feed offered and refused was determined on the same schedule of 0, 11, 21, 28 and 35 d and feed intake calculated as the difference between feed offered and refused for the 0 to 28 d pre-HS period, the 28 to 35 d HS period and the entire 0 to 35 d period. Body weight gain and feed intake by pen along with mortality weights were used to calculate mortality corrected feed conversion ratio (**FCRm**) by adding the pen mortality body weight gain to pen bird body weight gain. At 35 d of age, 5 broilers per pen were randomly selected, euthanized and defeathered. Dual X-ray absorptiometry (**DXA**) with a Lunar Prodigy machine (GE Lunar, GE Healthcare, Waukesha, WI) was utilized to measure fat and protein content of carcasses. Extra chicks that were not selected for the experiment at d 0 were euthanized and scanned for DXA as baseline measurement of chick body composition. Protein and fat accretion was calculated by the difference in protein and fat from 0 to 35 days by day.

### Cloacal Temperature and Panting

Cloacal temperature was measured at 28 and 35 d of age from 5 randomly selected broilers per pen. Cloacal temperatures were measured by inserting a thermometer (DeltaTrak MDL11064) exactly 2 cm into the cloaca. Individual cloacal temperatures were used as subsamples and averaged to determine a pen value for statistical analysis. Panting observations were performed by the same observer at 32 and 33 d of age on all broilers within the pen 2 h after heat stress was initiated and 1 h after heat stress was concluded. The observation was done on a scan sampling basis where the frequency of birds panting was observed. Panting was defined as an open beak with an abnormally rapid respiration rate. Non-panting was defined as a closed beak with normal respiration rate (Mack et al., 2013).

### Oxidative Stress

One bird per pen was randomly selected on 28 and 35 d of age. These birds were euthanized 2 h after the initiation of HS and liver sample collected and flash frozen in liquid nitrogen. A 0.3 g frozen liver sample was thawed, homogenized in 3 mL of 0.9% PBS using a bench top homogenizer (Tekmar company, OH). Total glutathione (**TGSH**), Oxidized glutathione (**GSSG**) and reduced glutathione (**rGSH**) were measured using an enzymatic recycling method (Cayman Chemical Company, Ann Arbor, MI). The sample was then deproteinated, and then the GSH in the sample reacted with DTNB (5,5’-dithio-bis-2-nitrobenzoic acid) to produce the yellow colored 5-thio-2- nitrobenzoic acid (**TNB**). The mixed disulfide, GSTNB, was reduced by glutathione reductase to recycle the GSH and produce more TNB. The amount of TNB production was quantified colorimetrically using the Infinite M200 Pro (Tecan, Morrisville, NC) set at 410 nm. The TNB concentration was directly proportional to the concentration of TGSH in the sample. Addition of glutathione peroxidase caused GSH to be oxidized to GSSG. Quantification of GSSG, was accomplished by first adding 2-vinylpyridine to GSH and the same methods as above were used to quantify GSSG. One molecule of GSSG in the body gets converted to 2 rGSH so there is double the amount of rGSH compared to GSSG. Thus, the rGSH levels were calculated by substracting twice the GSSG concentration from the TGSH concentration. The rGSH to GSSG ratio were also calculated to determine the redox status since under normal conditions most of the glutathione would be in the rGSH form (Owen and Butterfield, 2010).

### Intestinal Permeability

Intestinal permeability was estimated using a Fluorescein Isothiocyanate dextran (**FITC-d**) model (Baxter et al., 2017). This model is based on the fact that a large molecule dextran is not easily absorbed into the body, especially through the enterocyte itself. When intestinal structure is compromised, dextran can leak into circulation so quantifying serum dextran using a dosed and labeled dextran molecule can be used as a proxy for intestinal permeability. On 28, 31, and 35 d of age, one broiler per pen was randomly selected and orally gavaged with 8.32 mg/kg of FITC-d dissolved in double distilled water 1 h before blood sample collection. Blood was collected from the brachial vein transferred to serum tubes and allowed to clot for 4 h at room temperature under dark conditions. Serum was isolated from the blood by centrifuging tubes at 2,000 × *g* for 10 min at 4°C. Serum was then removed and diluted 1:5 in sterile 0.9% saline to a total volume of 100 μL in 96-well flat bottom black plate. The serum was analyzed for FITC-d at an excitation wavelength of 485 nm and an emission wavelength of 528 nm using multimode plate reader (Infinite M200 Pro, Tecan). Serum fluorescent concentrations were then determined using a standard curve of FITC-d sera generated by direct addition of FITC-d into sera of control chickens from the same experiment that had not received FITC-d.

### Statistical Analysis

Data were analyzed as a 2 × 2 factorial with HS and SAA as the main effects in JMP 14. The normal distribution of the data was verified by assessing the normal quantile plots in JMP 14. Student's *t* test was used to separate significant least squares means with the probability set at *P* ≤ 0.05. Treatments were randomly allotted among blocks within rows that were used as the random variable in the analyses. All data were analyzed based on the pen level experimental unit. The BWG at 0 to 28 d was added to our statistical model as a covariate for 28 to 35 BWG analyses.

## RESULTS AND DISCUSSION

### Broiler Performance and Body Composition

The analyzed SAA were similar to formulated values in the grower and finisher diet phases but were lower than expected in the methionine supplemented diet in the starter phase (Table 1). Nevertheless, there was still an 0.07% increase in SAA with the supplemented group compared to nonsupplemented group. There was no difference in initial BW (*P* > 0.05) at placement and chicks averaged 43.5 g/bird. No interactions were observed between HS and dietary SAA for live performance or body composition (*P* > 0.05; Tables 2 and 3). Dietary SAA had no effect on the performance of broilers regardless of heat treatment. Previous experiments have concluded that when diets are formulated to be in excess of SAA requirement for maximum growth in TN conditions, there is no additional improvement in growth observed of broilers subjected to HS (Willemsen et al., 2011; Liu et al., 2019; Zeitz et al., 2020). From hatch to 28 d of age, (the period before HS) birds raised in the HS room resulted in a 40 g decrease in BWG and a 6-point worsening of FCRm (*P* ≤ 0.05). This result was unexpected and suggests a room effect. As a method to reduce the room effect, 28-d body weight was used as a covariate for analysis of the 28 to 35 d body weight data. As expected, broilers that were subjected to elevated temperature had reduced BWG and worsened FCR (*P* ≤ 0.01) from 28 to 35 d and 0 to 35 d compared to the broiler raised under TN conditions. Although this is consistent with previous research that demonstrated that HS resulted in a reduction in overall growth and feed efficiency (Deeb and Cahaner, 2002; Niu et al., 2009; Imik et al., 2012; Sohail et al., 2012) the previous room effect must also be considered when interpreting these data. As expected with the reduced BW, HS resulted in reduced protein and fat accretion from 0 to 35 d of age compared to broilers that were raised under TN conditions (*P* ≤ 0.01). Arbor Acre broiler chickens subjected to a cyclic HS (23.9 to 35°C) from d 28 to 49, observed a decrease in breast weight and abdominal fat in the HS group compared to the TN broilers at 49 d of age (Smith, 1993). As with broiler performance, lean or fatty tissue accretion was not altered by SAA supplementation (*P* > 0.05).

**Table 2:** The effect of sulfur amino acids (SAA) supplementation on body weight gain and mortality corrected feed conversion ratio (FCRm) on broilers over the 0 to 35 d period when exposed to heat stress from 28 to 35 d.^1^

Temperature^2^	Diet	Body weight gain			FCRm (g:g)		
		0 to 28	28 to 35	0 to 35	0 to 28	28 to 35	0 to 35
		^________________^(g)^__________________^			^_________^ (g feed/g gain) ^_________^		
TN		1,388^a^	741^a^	2,129^a^	1.561^b^	1.762^b^	1.602^b^
HS		1,348^b^	649^b^	1,997^b^	1.620^a^	1.866^a^	1.661^a^
Pooled SEM		13	17	23	0.032	0.034	0.022
	Normal DSAA	1,386	689	2,075	1.580	1.841	1.651
	130% DSAA	1,350	701	2,051	1.609	1.782	1.623
Pooled SEM		13	17	23	0.032	0.034	0.022
*P* value^c^							
Temperature		**0.05**	**≤ 0.01**	**≤ 0.01**	**0.02**	**≤ 0.01**	**≤ 0.01**
Diet		0.10	0.72	0.16	0.23	0.31	0.41
Temperature × Diet		0.20	0.88	0.11	0.79	0.26	0.55

1 Values are means from 9 replicate pens from each interaction or 18 per main effect.

2 TN = continuous 22 to 24°C; HS received 33°C for 6 h and 27.7°C for the remaining 18 h daily.

a-b Values in a column without common superscript letter are different (*P* ≤ 0.05).

c Boldface indicates significant *P*-value.

**Table 3:** The effect of sulfur amino acids (SAA) supplementation on the overall lean and fat tissue accretion from 0 to 35 d of age of broiler chickens exposed to heat stress from 28 to 35 d in both experiments.^1^

Temperature^2^	Diet	Protein (g/day)	Fat (g/day)
TN		56.6^a^	9.8^a^
HS		52.0^b^	8.7^b^
Pooled SEM		1.31	0.12
	Normal DSAA	53.8	9.3
	130% DSAA	54.8	9.1
Pooled SEM		1.31	0.12
*P* value^d^			
Temperature		**0.02**	**≤ 0.01**
Diet	0.57	0.31	
Temperature × Diet	0.57	0.59	

1 Values are means from five birds per pen from 9 replicate pens from each interaction or 18 per main effect.

2 TN = continuous 22 to 24°C; HS received 33°C for 6 h and 27.7°C for the remaining 18 h daily.

a-b Values in a column without common superscript letter are different (*P* ≤ 0.05).

d Boldface indicates significant *P*-value.

### Cloacal Temperature and Panting

Heat stress was confirmed by measuring broiler cloacal temperatures 2 h after the initiation of HS on 28, 31, and 35 d of age. No interactions were observed between HS and SAA on cloacal temperature or panting (*P* > 0.05; Table 4). Broilers that were subjected to HS had increased cloacal temperatures 2 h after the initiation of HS at 28, 31, and 35 d of age compared to broilers under TN temperatures (*P* ≤ 0.01). The incidence rate of panting in broilers that were subjected to HS was increased to 96 ± 2.4% two hours after HS exposure compared to 6.66 ± 2.2% broilers that were under TN conditions on both 32 and 33 d (*P* ≤ 0.01). The panting effect was still observed one hour after the conclusion of HS, but to a lesser extent at 31% incidence (*P* ≤ 0.01). Previous experiments have reported consistent HS responses on body temperature and panting (Felver-Gant et al., 2012; Mack et al., 2013; Zeitz et al., 2020).

**Table 4:** The effect of sulfur amino acids (SAA) supplementation on cloacal temperature and panting (measured 2 h after daily heat exposure on d 28, 31, and 35 and 1 h after end of heat stress [HS] for panting) broilers exposed to heat stress from 28 to 35 d of age.

Temperature program^2^	Diet							
		Cloacal temperature			Panting			
		28	31	35	32 d	32 d 1 h after HS	33d	33 d 1 h after HS
		^_______________^°C ^________________^			^_______________________^ % ^_______________________^			
TN		41.9^b^	41.9^b^	41.8^b^	6.66^b^	3.52^b^	2.13^b^	3.62^b^
HS		43.7^a^	43.3^a^	43.0^a^	95.53^a^	31.60^a^	91.57^a^	38.46^a^
Pooled SEM		0.07	0.06	0.04	2.51	2.68	1.72	2.72
	Normal DSAA	42.9	42.6	42.6	55.36^a^	18.84	47.86	19.00
	130% DSAA	42.7	42.5	42.5	46.82^b^	16.28	45.84	23.07
Pooled SEM		0.07	0.06	0.04	2.51	2.68	1.72	2.72
*P* value^c^								
Temperature		**≤ 0.01**	**≤ 0.01**	**≤ 0.01**	**≤ 0.01**	**≤ 0.01**	**≤ 0.01**	**≤ 0.01**
Diet		0.19	0.21	0.64	**0.02**	0.50	0.41	0.30
Temperature × Diet		0.15	0.65	0.64	0.91	0.75	0.37	0.86

^1^Values are means from five birds per pen or whole pen from 9 replicate pens from each interaction or 18 per main effect.

2 TN = continuous 22 to 24°C; HS received 33°C for 6 h and 27.7°C for the remaining 18 h daily.

a-b Values in a column without common superscript letter are different (*P* ≤ 0.05)

c Boldface indicates significant *P*-value.

Supplementation of diets with SAA reduced panting 2 h after the initiation of HS on 32 d of age (*P* ≤ 0.05); however, this effect was inconsistent and not shown on 33 d or after the conclusion of HS on either day (*P* > 0.05). There is no clear mechanism of how SAA can reduce panting, but this inconsistent response has been noted previously. Cobb 500 male broilers subjected to a moderate continuous HS at 27.4°C showed reduced respiration rate with methionine supplementation compared to broilers with no additional methionine supplementation at 4 but not 3 or 5 wk of age (Zeitz et al., 2020).

### Oxidative Stress

**Figure 1:**
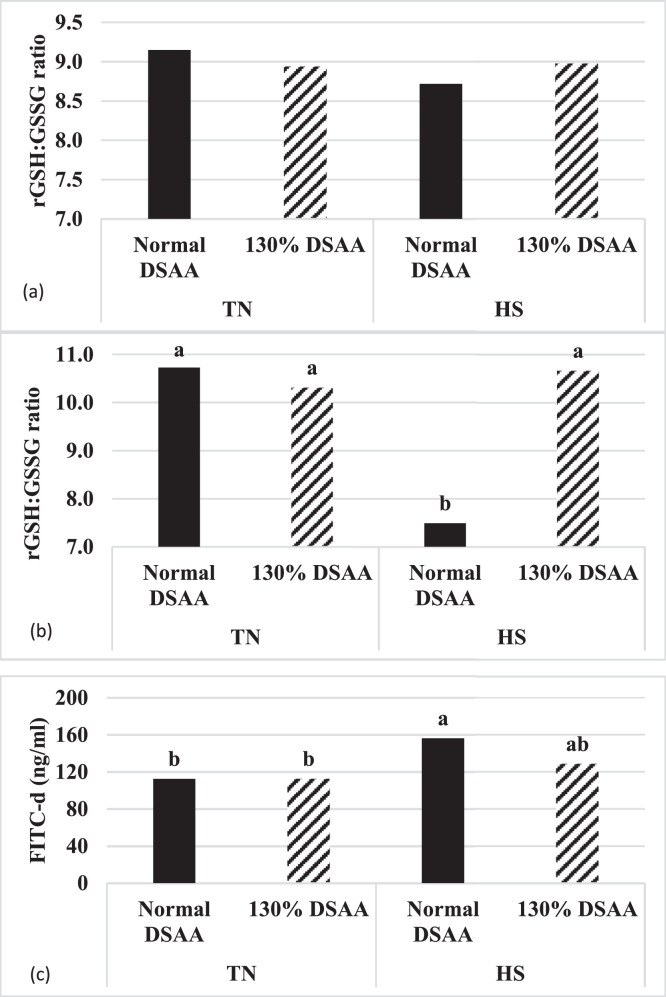
The effect of a SAA supplementation on (A) reduced glutathione to oxidized glutathione ratio in the liver at 28 d of age sampled 2 h after the initiation of heat stress (*P* = 0.08; SEM = 0.11), (B) reduced glutathione to oxidized glutathione ratio in the liver at 35 d of age sampled 2 h after the initiation of heat stress (*P* = 0.05; SEM = 0.80), and (c) FITC-d concentration in the serum at 28 d of age 2 h after the initiation of heat stress (*P* = 0.05; SEM = 8) of broiler chickens exposed to heat stress from 28 to 35 d. Abbreviation: SAA, sulfur amino acids.

A trend (*P* = 0.08) was observed for an interaction during the acute phase of HS at 28 d between environmental temperature and SAA supplementation on the rGSH:GSSG ratio (Figure 1A; Table 5). Liver tissue of broilers subjected to HS had a reduced rGSH:GSSG ratio compared to livers of broiler under TN temperature indicating a reduction in redox potential. After just 2 h of HS, supplementation of SAA to broilers partially improved the redox status on d 28 (Figure 1A; *P* = 0.08). After birds were exposed to cyclic HS for 7 d, the effects of increased dietary SAA were more apparent in reducing the ratio of rGSH:GSSG in broiler subjected to HS compared to TN exposed broilers (Figure 1B; *P* ≤ 0.05). These results are consistent with a previous report with male Ross broilers subjected to a continuous HS at 32°C from 2 to 6 wk of age. The authors reported a reduction in redox capacity with HS compared to TN while showing mitigation of this reduction with additional methionine supplementation (Willemson et al., 2011). Cobb 500 broilers subjected to acute HS at 38°C for 24 h at 21 d of age reported an increase in glutathione peroxidase activity with supplementation of excessive methionine during heat stress (Del Vesco et al., 2015). Glutathione peroxidase catalyzes the reduction of free radical damage. This implies that SAA not only reduces oxidative stress but also likely improves the activity of key enzymes needed for this process. Male Cobb 500 broilers subjected to a moderate continuous HS at 27.4°C from 3 to 5 wk of age found no effect on the rGSH:GSSG ratio but found that the additional supplementation of SAA increased the hepatic concentration of both rGSH and GSSG (Zeitz et al., 2020). These data might suggest that higher temperatures are required to generate an oxidative response in broilers, but supplemental SAA are an important precursor to maintain oxidative balance.

**Table 5:** The effect ofsulfur amino acids (SAA) supplementation on hepatic GSSG, rGSH concentrations (measured 2 h after daily heat exposure on d 28 and 35) of broilers exposed to heat stress from 28 to 35 d of age.^1^

Temperature program^2^	Diet						
		28 d (Acute)			35 d (Chronic)		
		GSSG	rGSH	rGSH:GSSG	GSSG	rGSH	rGSH:GSSG
		^_________^ nmol/g ^_________^			^_________^ nmol/g ^_________^		
TN		418	3,779	9.07	378^b^	4,138	11.81
HS		417	3,689	8.86	538^a^	4,514	8.91
Pooled SEM		5.5	61	0.091	28	154	0.743
	Normal DSAA	417	3,722	8.93	482	4,253	9.96
	130% DSAA	418	3,746	8.96	434	4,399	10.75
Pooled SEM		5.5	61	0.091	28	154	0.743
*P* value^c^							
Temperature program		0.91	0.91	0.15	**≤ 0.01**	0.09	0.01
Diet		0.86	0.86	0.83	0.23	0.51	0.46
Temperature × Diet		0.44	0.44	0.08	0.13	0.17	**0.05**

1 Values are means from one bird per pen from 9 replicate pens from each interaction or 18 per main effect.

2 TN = continuous 22 to 24°C; HS received 33°C for 6 h and 27.7°C for the remaining 18 h daily.

a-b Values in a column without common superscript letter are different (*P* ≤ 0.05).

c Boldface indicates significant *P*-value.

### Intestinal Permeability

Two hours after first initiation of HS on 28 d of age, there was an interaction observed between temperature and SAA supplementation (Figure 1C; *P* ≤ 0.05). Heat stress without additional dietary SAA resulted in an increase of serum FITC-d compared to TN birds that was mitigated by the higher dietary SAA in the HS group. This interaction suggests a more permeable intestine generated by the HS that was ameliorated by the increased supplementation of SAA. Broilers subjected to HS had higher serum FITC-d concentrations compared to TN group at 31 d of age regardless of SAA supplementation (Table 6; *P* ≤ 0.05) indicating compromised intestinal structure and a “leaky” gut. After the broilers had been subjected to the cyclic HS for 7 d, the serum FITC-d concentrations similar to those of the TN groups (*P* > 0.05). These results are consistent with a previous experiment investigating the effects of an acute 36°C heat stress for 2 h which resulted in an increase in serum FITC-d concentration in several genetic lines of broilers compared to TN (Tabler et al., 2020). This increase in FITC-d was related to downregulation of several tight junction proteins in both the jejunum and ileum. A continuous 35°C HS treatment was provided to 21 to 42 d of age Cobb 500 broilers which resulted in serum FITC-d concentrations increasing with the HS at both 35 and 42 days of age (Ruff et al., 2020). The disruption of tight junction proteins has been associated with secretion of proinflammatory cytokines into the intestinal tract which could cause reduced performance and efficiency (Bailey et al., 2011). Oxidative stress and the increase in free radicals have also been reported to disrupt these tight junctions in the intestine and increase intestinal permeability (Sanders et al., 2005).

**Table 6:** The effect of sulfur amino acids (SAA) supplementation on FITC-d in the serum (measured 2 h after daily heat exposure on d 28 and 35) of broilers exposed to heat stress from 28 to 35 d of age.^1^

Temperature^2^	Diet	FITC-d concentration		
		28 d	31 d	35 d
		^______________________________^ ng/mL ^_____________________________^		
TN		113	110^b^	117
HS		142	116^a^	121
Pooled SEM		6	2	3
	Normal DSAA	135	113	119
	130% DSAA	119	112	118
Pooled SEM		6	2	3
*P* Value^c^				
Temperature		≤ 0.01	**0.05**	0.34
Diet	0.05	0.67	0.81	
Temperature × Diet	**0.05**	0.73	0.87	

1 Values are means from one bird per pen from 9 replicate pens from each interaction or 18 per main effect.

2 TN = continuous 22 to 24°C; HS received 33°C for 6 h and 27.7°C for the remaining 18 h daily.

a-b Values in a column without common superscript letter are different (*P* ≤ 0.05).

c Boldface indicates significant *P*-value.

In conclusion, methionine supplemented to 130% of the SAA requirement did not improve performance parameters of broilers exposed to a cyclic heat stress. However, SAA supplementation was able to improve the antioxidant function of broilers exposed to HS as demonstrated by the increase of the ratio of rGSH:GSSG. This response is thought to be mediated by an increase in the production of the antioxidant glutathione starting the acute response to HS and showing significant responses after chronic HS exposure. The supplementation of methionine to increase dietary SAA reduced the intestinal permeability of broilers during the acute HS phase. In this experiment, the effects of HS on oxidative stress were more prominent over time but the effects on intestinal permeability were more pronounced with first acute exposure and diminished overtime.
